# Blood markers of oxidative stress in Alzheimer's disease

**DOI:** 10.1111/j.1582-4934.2012.01585.x

**Published:** 2012-09-26

**Authors:** Alice Skoumalová, Jakub Hort

**Affiliations:** a Department of Medical Chemistry and Biochemistry, Charles University in Prague 2^nd^ Faculty of MedicinePrague, Czech Republic; b Memory Disorders Clinic Department of Neurology, Charles University in Prague 2^nd^ Faculty of Medicine and University Hospital MotolPrague, Czech Republic; c International Clinical Research Center, St.Anne's University Hospital BrnoBrno, Czech Republic

**Keywords:** Alzheimer disease, blood markers, oxidative stress, lipid peroxidation

## Abstract

Alzheimer′s disease (AD) represents a highly common form of dementia, but can be diagnosed in the earlier stages before dementia onset. Early diagnosis is crucial for successful therapeutic intervention. The introduction of new diagnostic biomarkers for AD is aimed at detecting underlying brain pathology. These biomarkers reflect structural or biochemical changes related to AD. Examination of cerebrospinal fluid has many drawbacks; therefore, the search for sensitive and specific blood markers is ongoing. Investigation is mainly focused on upstream processes, among which oxidative stress in the brain is of particular interest. Products of oxidative stress may diffuse into the blood and evaluating them can contribute to diagnosis of AD. However, results of blood oxidative stress markers are not consistent among various studies, as documented in this review. To find a specific biochemical marker for AD, we should concentrate on specific metabolic products formed in the brain. Specific fluorescent intermediates of brain lipid peroxidation may represent such candidates as the composition of brain phospholipids is unique. They are small lipophilic molecules and can diffuse into the blood stream, where they can then be detected. We propose that these fluorescent products are potential candidates for blood biomarkers of AD.

Introduction
Biochemical processes related to underlying AD pathology
Lipid peroxidation in AD
Blood markers of lipid peroxidation in AD
Protein oxidation in AD
Blood markers of protein oxidation in AD
Nucleid acid oxidation in AD
Blood markers of DNA/RNA oxidation in AD
Blood antioxidants in AD
Are blood markers of oxidative stress specific for AD?
Specific fluorescent products of lipid peroxidation
Conclusion

## Introduction

Alzheimer′s disease (AD) is a progressive neurodegenerative disorder of the brain that is characterized by a loss of neurons because of extracellular accumulation of amyloid beta (Ab) and intracellular hyperphosphorylation of the tau protein. However, a proper pathophysiological mechanism of the disease's evolution is very complex and involves many biochemical mechanisms. As, at the start, AD typically affects the hippocampus and adjacent structures, memory deficits are typically among the earliest and most pronounced signs of AD. When pathological changes spread beyond the hippocampus, other cognitive areas also become affected. The majority of AD cases are sporadic and typically occur in age groups over 65 years.

Definitive diagnosis requires histological analyses of the brain tissue providing senile plaques and tangles. Clinical diagnosis is based on clinical criteria, which were first consistently postulated in 1984 [1]. These criteria were recently challenged by new criteria [2–4], which put more stress on the early diagnosis of AD and the introduction of biomarkers. There is an effort to distinguish the healthy elderly and those individuals at risk of AD who carry risk factors; individuals with an amyloid burden in the brain, but no clinical symptoms (preclinical AD); individuals with the first clinical symptoms (prodromal AD, which usually refers to mild cognitive impairment – MCI) as well as to differentiate AD dementia from other disorders (*e.g*. vascular dementia, Lewy body dementia, frontotemporal lobe dementiaand Parkinson′s disease).

The effects of drugs favourably influencing the pathological processes of AD are expected to be most effective in the early stages rather than at the stage of dementia. Consequently, there is an increasing effort to find new biomarkers of AD as they could make earlier diagnosis possible in a clinical setting. They should be highly specific to AD and sensitive to changes, especially in the early stages of the disease.

There are several candidate biomarkers for the diagnosis of AD reflecting structural changes (MRI volumometry), metabolic changes (the uptake of radiolabelled substances measured using PET or SPECT) or new sensitive neuropsychology tests [5–7]. These biomarkers may differ in sensitivity, specificity, cost-effectiveness, invasivity, logistical and technical demands. For example, Pitsburg compound B (PIB) can image amyloid *in vivo*. However, it is not routinely available. Furthermore, PIB pozitive findings are present in approximately 20–30% of healthy elderly and 50–60% of MCI patients [8].

Studies on biochemical markers for the diagnosis of AD and MCI in cerebrospinal fluid (CSF) and blood are based on the detection of inflammatory proteins, markers of cholesterol homeostasis, oxidative stress, or related to characteristic pathological alterations in AD [9]. The combination of detecting the tau protein, phosphorylated tau protein and Ab in CSF has a sensitivity and specificity of about 90% for AD diagnosis. On the other hand, the invasivity of lumbar puncture and logistical issues related to this procedure do not allow its use in routine screening of patients. An ideal early detection of AD and other types of dementia would require simple, non-invasive and inexpensive diagnostic tests. However, to date, no validated diagnostic marker in peripheral blood for early diagnosis of AD has been found. For example, there is an effort to use blood Ab for diagnosis. Nevertheless, assessment of Ab in plasma brought contradictory results as Ab binds to plasma proteins. This may be derived from peripheral tissues and does not necessarily reflect brain metabolism [10].

Oxidative stress accompanies pathological changes in AD and MCI and is considered to be a crucial upstream factor in the pathogenesis of the disease [see, for example, review [11]]. Products of free radical damage, such as aldehydes or lipid hydroperoxides, may diffuse into the blood where they can be detected. Moreover, it has been found that blood-brain barrier (BBB) permeability significantly increases in both AD and vascular dementia as compared with ageing controls [12, 13]. Consequently, products of oxidative stress represent potential biomarkers in blood for diagnosis of AD. On the other hand, other diseases accompanied by free radical production, such as diabetes or cardiovascular disease, may influence the presence of free radical products in the blood. This could explain the fact that the results of oxidative stress markers in the blood in AD are not consistent in various studies.

In this review, we discuss the presence of oxidative stress markers in the blood in AD and MCI as well as their specificity. Moreover, we focus on specific fluorescent products of lipid peroxidation in AD and their potential use for diagnosis.

## Biochemical processes related to underlying AD pathology

The pathophysiology of AD is a very complex process, which includes many pathological changes. Accumulation of Ab is upstream to tau pathology [14]. Many processes occur before Ab misfolding and many others occur in parallel with these processes. Ab is a product of Ab precursor protein (APP), which is enzymatically cleaved by α-, β- and γ-secretases to release several forms of Ab peptides. So called amyloidogenic processing involves β- and γ-secretases, whose catalytic subunits are known as presenilins. Although the physiological role of APP and Ab peptides remains unclear, there are links between Ab and the pathophysiology of AD. The formation of senile plaques, composed predominantly of Ab peptides, is one of the hallmarks of the disease.

Hyperphosphorylation of tau proteins is also involved in the pathogenesis of AD. The main function of tau proteins is to promote neuronal microtubule stability and assembly. They are involved in promoting microtubule nucleation, growth and bundling. The accumulation of hyperphosphorylated tau proteins in AD, which is the result of an imbalance in the kinase and phosphatase activities, leads to the formation of neurofibrillary tangles.

Another event, which is discussed as a consequence of AD, is an overproduction of free radicals. Oxidative stress is present in AD as a result of Ab misfolding, which is accompanied by the activation of microglia. The enzyme NADPH oxidase, localized in the microglia membranes, is activated in the brains of AD patients resulting in the production of free radicals [15]. Furthermore, microglial activation occurs early in AD development [16].

Ab peptides also represent important sources of free radicals in AD. It has been found that Ab directly generates free radicals for which methionine, at the position of 35, is responsible [17]. Moreover, Ab binds redox active metals [18], which play an important catalytic role in the production of free radicals. Fe^2+^ is involved in the generation of the hydroxyl radical, one of the most toxic oxidants with the potential to initiate lipid peroxidation of fatty acids. Fe^2+^ concentration in the brain in AD is increased [19].

Furthermore, it has been found that oxidative stress in the brain even precedes the formation of senile plaques and tangles. For example, lipid peroxidation products accumulate in neurons with no other signs of AD pathology [20]. Moreover, the generation of 8-hydroxyguanosine and nitrotyrosine, products of free radical damage to RNA and proteins, in the cytoplasm of neurons from Down's syndrome patients appears decades prior to Ab accumulation [21, 22]. Furthermore, lipid peroxidation occurs before the formation of Ab plaques in transgenic mouse models of AD [23]. These findings support the hypothesis that free radical damage is present in the brain in the preclinical stage of AD.

## Lipid peroxidation in AD

Brain tissue is rich in phospholipids, which is crucial to the processes of neural transmission. Brain phospholipids contain a high percentage of polyunsaturated fatty acids (PUFA), particularly docosahexaenoic acid with six double bonds and arachidonic acid with four double bonds. In the case of increased free radical production, PUFA are primarily attacked because the presence of conjugated double bonds facilitates the process of lipid peroxidation. As a result of free radical overproduction, there is a reduced content of PUFA in the brain in AD [24].

Products of lipid peroxidation, lipid hydroperoxides, are unstable and in the presence of iron are non-enzymatically decomposed to a number of varying products, such as aldehydes malondialdehyde (MDA) and 4-hydroxynonenal (4-HNE), ketones, epoxides and hydrocarbons. Increased levels of MDA and 4-HNE in the brain in AD and MCI have been confirmed by several studies [25–28]. Aldehydes formed during lipid peroxidation of brain PUFA can diffuse from the primary sites and be used as markers of oxidative stress.

Another outcome of lipid peroxidation is the formation of isoprostanes. They are prostaglandin-like compounds formed from PUFA with at least three double bonds, including arachidonic and docosahexaenoic acid. Isoprostanes are produced *in vivo* by peroxidation of phospholipids non-enzymatically, in contrast with prostaglandins generated by enzymes, and their measurement is probably the best currently available assay of lipid peroxidation. F2-isoprostanes (F2-IsoPs) are formed from arachidonic acid *via* esterification with phospholipids followed by hydrolysis. In AD, increased levels of F2-IsoPs were detected in cerebrospinal fluid (CSF) [29, 30]. The amount of F2-IsoPs in the ventricular fluid correlates negatively with brain weight [31]. Furthermore, the amount of F2-isoprostanes is increased in MCI [32].

Compounds structurally related to isoprostanes are F4-isoprostanes (F4-IsoPs), products of radical peroxidation of docosahexaenoic acid, a highly prevalent PUFA in the brain. As a result of six double bonds, docosahexaenoic acid is even more prone to free radical attack than arachidonic acid. Therefore, the detection of its peroxidative products is an important marker of brain oxidative damage and useful in neurodegenerative diseases. The level of F4-IsoPs was found to have increased in CSF in AD compared with controls [33].

## Blood markers of lipid peroxidation in AD

**Fig 1:**
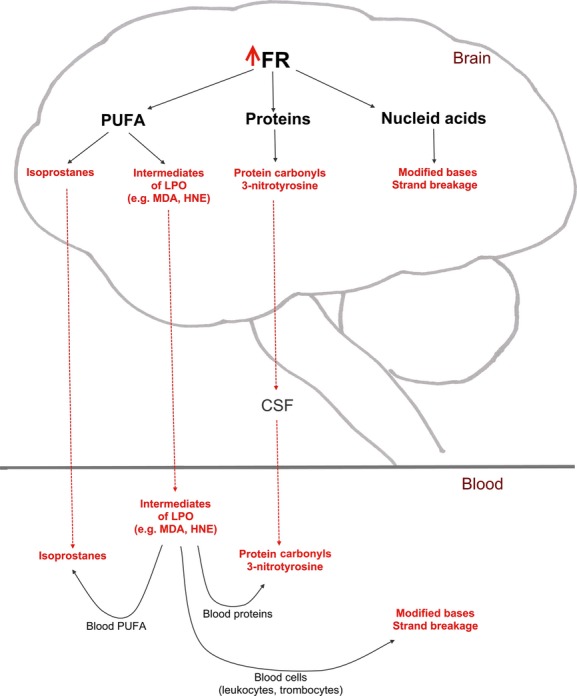
The origin of blood oxidative stress markers in AD. Increased production of free radicals in the brain in AD results in lipid peroxidation, protein and nucleic acid oxidation. Products of lipid peroxidation (*e.g*. isoprostanes, lipid hydroperoxides and aldehydes) are small, lipophilic compounds that can diffuse to the blood where they can be detected. Products of protein oxidation (protein carbonyls, 3-nitrotyrosine) can be secreted to the CSF, which is absorbed in the blood. Oxidative stress products may also be generated directly in the blood. Reactive intermediates of lipid peroxidation may attack blood proteins and PUFA or cause damage to nucleic acids in blood cells. FR, free radicals; CSF, cerebrospinal fluid; PUFA, polyunsaturated fatty acids; LPO, lipid peroxidation; MDA, malondialdehyde; HNE, 4-hydroxynonenal.

Lipid peroxidation intermediates formed in the brain may travel through the BBB as they are small, lipophilic molecules and may reach the blood (see Fig. 1). Much research has focused on the determination of MDA or 4-HNE in the blood and their potential use as markers of the brain oxidative stress in AD.

MDA arises largely from the peroxidation of PUFA. It exists either in a free form or in bound to proteins. Free MDA *in vivo* is rapidly metabolized in tissues. A number of studies document elevated levels of MDA in AD and MCI in the plasma/serum [34–42]. Increased concentrations of thiobarbituric reactive substances in the serum [43] or erythrocytes [44, 45] of patients with AD were also documented. In contrast, there are studies that did not find differences in the concentration of MDA between AD patients and controls [46–51].

Another important product of lipid peroxidation, 4-HNE, is formed during the peroxidation of linoleic and arachidonic acid. It is one of several unsaturated aldehydes generated during lipid peroxidation. In the plasma of AD patients, the amount of 4-HNE was increased compared with controls [50, 52, 53].

The determination of MDA and 4-HNE as a measure of lipid peroxidation should be interpreted with caution because of their reactivity with proteins and rapid metabolism. On the other hand, isoprostanes represent the best available biomarker of lipid peroxidation nowadays. Most study has been carried out on the F2-isoprostanes, which arise from arachidonic acid, but some data are available on isoprostanes derived from docosahexaenoic acid (F4-isoprostanes). Isoprostanes were analysed in body fluids as potential markers of oxidative stress in AD and MCI. Levels of F2-IsoPs were elevated in the blood, CSF and urine in AD [30] and in MCI [54]. Furthermore, their concentrations correlate with measures of cognitive and functional impairment in AD patients [30]. Another study also shows higher F2-IsoPs in serum in AD [42]. On the other hand, there are studies that did not find differences in blood isoprostanes between AD patients and controls [29, 55, 56]. Plasma and urine F2 and F4-IsoPs do not reflect central nervous system levels [57]. Within the MCI and AD groups, F2-IsoPs levels did not correlate with the duration of memory impairment or with cognitive test scores [55]. Associations between levels of isoprostanes and tocopherols and the development of AD were not confirmed in a longitudinal study [58].

Peripheral cells of patients with AD were also studied and analysed for markers of lipid peroxidation. Fibroblasts and lymphoblasts from patients with familial AD carrying amyloid precursor protein and presenilin-1 gene mutations showed an increase in MDA and 4-HNE. However, in the same study, these products in lymphoblasts from patients affected by sporadic AD were virtually indistinguishable from the basal values of normal controls [41].

## Protein oxidation in AD

Free radical compounds may further attack proteins. Damage to proteins can occur either by direct attack of free radicals or secondarily by end-products of lipid peroxidation, such as isoketals, MDA and 4-HNE. Increased levels of protein carbonyls, markers of oxidative damage to proteins, have been documented in the AD brain by several studies [27, 59].

Reactions of various reactive oxygen and nitrogen species with tyrosine lead to the production of 3-nitrotyrosine and dityrosine. It has been documented that the concentration of 3-nitrotyrosine was increased in the CSF of persons with AD. The Mini-Mental State Examination (MMSE) score correlated negatively with 3-nitrotyrosine residue concentration in CSF [60]. Furthermore, protein nitration represents an early event in the pathogenesis of AD. The level of total protein nitration in brain samples from persons with MCI compared with that in healthy controls was higher in the inferior parietal lobule and hippocampus [61].

## Blood markers of protein oxidation in AD

The determination of oxidatively modified proteins in the blood represents another possibility to measure oxidative stress in various diseases including AD. As concerns AD, products of protein oxidation can originate from the brain and reach the blood *via* the BBB, or blood proteins can be oxidized directly in the bloodstream. In the second case, blood proteins are attacked by lipid radicals and other lipophilic compounds produced during lipid peroxidation in the brain and crossing the BBB (see Fig. 1). Blood proteins can be oxidized randomly or some of them can be more sensitive to oxidative damage. Oxidatively modified proteins in the blood can be determined either as total protein carbonyls or by a proteomic approach as specific protein modifications.

Total oxidized proteins were analysed in serum in AD and MCI and several studies have found that they increase. For example, the levels of carbonyl proteins were significantly higher in the AD/MCI group (approximately three times) compared with controls [62]. Other researchers analysed protein carbonyls in plasma in patients with MCI and AD. The results showed an increase in protein modification in AD and MCI patients compared with age-matched control individuals[63]. Furthermore, the levels of oxidatively modified proteins were examined in the blood from AD patients, non-AD controls and AD relatives. Statistically significant elevations of total oxidized proteins were observed in both AD individuals and AD relatives when compared with non-AD controls [64]. Nevertheless, results on the products of protein oxidation in the blood in AD are conflicting as no differences in total plasma protein carbonyl content have been documented by other studies [42, 51, 65].

Analyses of specific blood protein oxidations may be more useful in searching for biomarkers of AD. A proteomic approach was employed to elucidate possible specific oxidative modifications of plasma proteins in AD. Proteins, which showed specific oxidation in AD were identified as isoforms of human transferrin, hemopexin and alpha-1-antitrypsin [66]. Another study showed a specific oxidation of the fibrinogen gamma-chain precursor protein and of the alpha-1-antitrypsin precursor in the plasma in AD [67]. Furthermore, an increased content of carbonyl proteins and dityrosine in immunoglobulin G was documented in AD [51] and the content of carbonyl groups was elevated in LDL [65].

3-nitrotyrosine represents a useful marker of protein oxidation. However, in contrast with its elevated levels in the brain, there is only one study documenting increased levels of 3-nitrotyrosine in the plasma of AD patients [53].

## Nucleid acid oxidation in AD

Oxidative damage to DNA can be measured as chemical modifications to the DNA bases or to deoxyribose. For example, oxidation of DNA may result in the formation of 8-hydroxy-2′-deoxyguanosine (8-OHdG). Oxidative DNA damage appears to occur continuously *in vivo*. However, it is exacerbated in diseases accompanied by oxidative stress. Levels of 8-OHdG in mitochondrial DNA isolated from the parietal cortex of AD patients were significantly (three times) increased compared with controls [68]. Oxidative modifications to RNA were detected by means of immunocytochemistry with results showing increased levels of 8-hydroxyguanosine (8-OHG) in the AD brain [69].

Another possible way to measure oxidative damage to DNA is by determining DNA strand breakage. It has been documented that the level of DNA breaks in cerebral cortex tissue samples from AD patients and controls obtained from rapid autopsies were twice as high in AD patients compared with controls [70].

## Blood markers of DNA/RNA oxidation in AD

Oxidative damage to DNA or RNA in blood cells in AD was monitored by several researchers. The most common method used for determining oxidative damage to DNA is the measurement of modified bases, most often the nucleoside 8-OHdG. A significantly higher concentration of 8-OhdG in lymphocytes occurred in AD patients compared with controls [71, 72].

The level of oxidative damage and repair capacity in peripheral lymphocytes of AD patients and of age-matched controls was determined. Statistically significant elevations of oxidized purines were observed in the nuclear DNA of peripheral lymphocytes from AD patients compared with age-matched control individuals [73]. Another study also demonstrated that AD was associated with elevated levels of oxidized pyrimidines and purines compared with age-matched control individuals [74].

Moreover, it has been documented that oxidative damage to DNA in the blood is an earlier event in the pathogenesis of AD. The study was performed to evaluate the level of oxidative DNA damage in two groups of MCI and AD patients compared with healthy controls. Data showed a significantly higher level of DNA damage in the leukocytes of AD and also of MCI patients compared with control individuals. Furthermore, the amount of oxidized DNA bases (both purines and pyrimidines) was significantly higher in the two groups of patients (AD and MCI) compared with controls [75].

To investigate oxidative damage to RNA in AD, the concentration of 8-OHG was measured in the CSF and serum of patients with AD and control individuals. The concentration of 8-OHG in the CSF in AD patients was approximately 5-fold than in controls. The concentration of 8-OHG in the CSF decreased significantly with the duration of the illness and the progression of cognitive dysfunctions. However, the concentration of 8-OHG in the CSF showed no correlation with that in serum in both the controls and AD patients. In addition, the concentration of 8-OHG in serum was not significantly altered in AD patients compared with that in controls, suggesting that the 8-OHG concentrations in the CSF do not reflect those in serum and may probably reflect those in the brain tissue [76].

## Blood antioxidants in AD

Levels of antioxidants in the blood were also analysed as a consequence of oxidative stress in AD as they are consumed in the case of free radical production. Vitamin E represents a major chain breaking antioxidant, which prevents peroxidation of PUFA in biological membranes. It is especially important for the brain, considering the high lipid content and high proportion of PUFA. Vitamins C and A are also involved in the metabolism of free radicals. A number of studies have shown reduced levels of vitamin E [37, 49, 51, 77–80], vitamin C [50, 51, 77–79] or vitamin A [37, 51, 77, 78, 80] in the plasma or serum in AD. This difference was found in AD patients under normal dietary circumstances, *i.e*. without any supplementation. Decreased vitamin E concentration in the plasma in AD correlates positively with its concentration in CSF [81]. However, some studies did not find differences in the levels of vitamin E [46], vitamin C [82] or vitamin A [49] in the plasma in AD.

Changes in other antioxidants in AD were also monitored in the plasma and erythrocytes. It has been found that total antioxidant capacity of the plasma was decreased in AD patients [43, 65, 83, 84] and negatively correlates with the duration of the disease [84]. These results are not in agreement with another study, in which total plasma antioxidant capacity in AD is the same as in controls [49]. Furthermore, there was a decreased ratio of reduced and oxidized glutathion, lower activities of glutathion peroxidase [63] and superoxide dismutase [85] in erythrocytes in AD, the latter only in women.

## Are blood markers of oxidative stress specific for AD?

It can be assumed from the above that oxidative stress in blood often accompanies AD and MCI. Many researchers found increased levels of oxidative stress markers or decreased levels of antioxidants in the blood in AD or MCI. These results are in agreement with the widespread belief that pathological processes in the brain in AD are accompanied by oxidative stress [11]. However, the results are not consistent among different reports, as documented in this review (see Table 1). There are studies that did not confirm elevated levels of free-radical products in the blood in AD and MCI patients. Therefore, it is difficult to identify AD specific markers. Furthermore, as oxidative stress also accompanies other diseases, such as diabetes, cardiovascular disease and other neurodegenerative diseases, the detection of products of free radical reactions may not be specific to AD and MCI. Consequently, the key question is whether the presence of markers of oxidative stress in the blood in AD and MCI is specific or not.

**Table 1:** Levels of oxidative stress markers in blood in AD

	Oxidative stress markers	Levels in blood	References
Lipid peroxidation markers	F2-isoprostanes	+	[30, 42, 54]
	Malondialdehyde	=	[29, 55–57]
	4-hydroxynonenal	+	[34–45]
		=	[46–51]
		+	[41, 50, 52, 53]
		=	[49]
Protein oxidation markers	Protein carbonyls	+	[38, 53, 62–64, 67]
	3-nitrotyrosine	=	[42, 51, 65]
		+	[53]
DNA/RNA oxidation markers	8-Hydroxy-2′-deoxyguanosine	+	[71–75]
	8-Hydroxyguanosine	=	[76]
Antioxidants	Vitamin E	−	[37, 49, 51, 77–80]
	Vitamin C	=	[46]
	Vitamin A	−	[50, 51, 77–79]
	Total plasma antioxidant capacity	=	[82]
	Glutathion	−	[37, 51, 77, 78, 80]
	Superoxide dismutase	=	[49]
		−	[43, 65, 83, 84]
		=	[49]
		−	[63]
		−	[85]

+, Increased levels; −, Decreased levels; =, No differences.

To answer this question, we can compare the presence of oxidative stress markers in different pathological states. There are studies that show the differences in oxidative stress products in various diseases. Discriminant functions constructed using biochemical markers of oxidative stress (superoxide dismutase, catalase, glutathione, thiobarbituric acid reactive substancesand antioxidant capacity of plasma) separated patients with AD from vascular dementia, but not patients with Parkinson′s disease from AD or vascular dementia [44]. Higher levels of MDA and lower levels of antioxidants were monitored in patients with AD compared with vascular dementia [39]. On the other hand, patients with AD and vascular dementia showed similar plasma levels of antioxidants and MDA as well as a similar IgG content of protein carbonyls and dityrosine [51]. Furthermore, levels of antioxidants were similarly reduced in AD, vascular dementia and Parkinson′s disease [77].

Inconsistent and conflicting data means these oxidative stress markers cannot be recommended for routine clinical use. Consequently, further studies referring to the sensitivity and specificity of existing markers are needed; in addition, a search for new candidates should be encouraged.

## Specific fluorescent products of lipid peroxidation

Another possible way to find a blood biomarker for AD is to concentrate on specific products. Intermediates of PUFA peroxidation may represent such compounds. As mentioned above, the lipid composition of brain PUFA is unique. There is a high content of highly unsaturated PUFA, particularly of docosahexaenoic acid. Consequently, the intermediates of brain lipid peroxidation represent specific products. Analyses of these products are complicated because the amount of lipid peroxides and unsaturated aldehydes formed during lipid peroxidation of PUFA increases with the number of carbons and double bonds. Nevertheless, several products of docosahexaenoic acid peroxidation were identified. For example, C16 and C20 hydroperoxides and the aldehydes 4-hydroxy-2-hexenal and 4-oxo-2-hexenal are among such products [86].

Intermediates of lipid peroxidation react with proteins and phospholipids to form fluorescent products, which have been named lipofuscin-like pigments (LFP), on the basis of the similarity of their fluorescence spectra with lipofuscin. Fluorescence analyses of lipid peroxidation products play an important role in the characterization of these complex mixtures and are useful in searching for AD specific markers.

As a result of their native fluorescence as well as the high sensitivity of fluorescence measurement, LFP detection can be used as an indicator of free radical damage in various biological systems [see, for example, [87]]. Fluorescence measurement is one of the methods analysing the end-products of lipid peroxidation. The application of special fluorescence techniques, such as tridimensional and synchronous spectra, enables both quantitative and qualitative changes in the composition of LFP, as a consequence of free radical damage, to be monitored. The levels of LFP were elevated in the brains of canine counterparts of AD compared with age-matched control animals as a result of lipid peroxidation [88].

**Fig 2:**
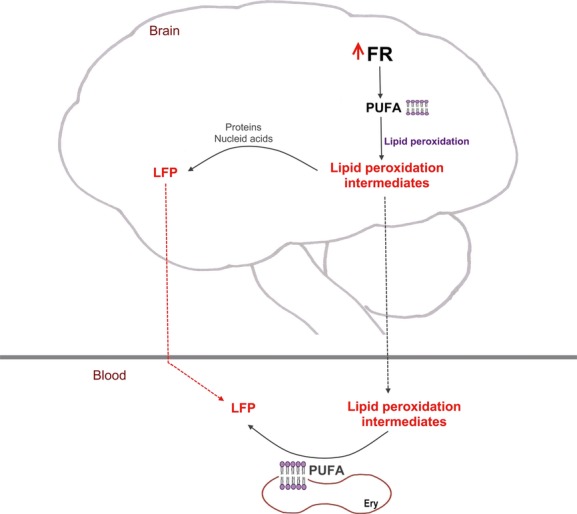
The origin of LFP in AD. Increased production of free radicals in the brain in AD results in the initiation of lipid peroxidation. Intermediates of lipid peroxidation (*e.g*. aldehydes) react with proteins and nucleic acids and generate LFP. Small, lipophilic intermediates of lipid peroxidation may diffuse across the BBB to the blood, attack erythrocyte PUFA resulting in the formation of LFP. The LFP formed in the brain may also diffuse to the blood. LFP, lipofuscin-like pigments; BBB, blood-brain barrier; PUFA, polyunsaturated fatty acids; FR, free radicals; Ery, erythrocytes.

Furthermore, specific fluorescence products formed in the brain in AD may diffuse across the BBB to the blood where they can be detected (see Fig. 2). There are studies on fluorescent analyses of LFP in the erythrocytes of AD. Levels of LFP were increased in the erythrocytes of dogs with the canine counterpart of AD compared with age-matched controls [88]. Moreover, the LFP was elevated in the erythrocytes of patients with AD. When the LFP in AD patients' erythrocytes was analyzsed using specific fluorescence measurements and separatedusing HPLC with a fluorescence detector, the presence of a specific fluorescent product was documented [89].

## Conclusion

AD can be detected before the onset of dementia syndrome at its prodromal or even preclinical stage. The proposed biomarkers have economic, logistical, or practical disadvantages as well as limited sensitivity and specificity. Blood tests would be practical if a reliable biomarker could be proven as a suitable tool to reflect the underlying AD pathology in individuals at risk of AD or those in the prodromal stage. Furthermore, the introduction of a new biomarker would be useful for differential diagnosis of dementia syndrome of various origins. Pathophysiological processes include lipid peroxidation and the formation of specific fluorescent products, which can diffuse into the blood. One perspective method is the detection of fluorescence products in the blood. However, ongoing research will have to validate these procedures.
